# Corra: Computational framework and tools for LC-MS discovery and targeted mass spectrometry-based proteomics

**DOI:** 10.1186/1471-2105-9-542

**Published:** 2008-12-16

**Authors:** Mi-Youn Brusniak, Bernd Bodenmiller, David Campbell, Kelly Cooke, James Eddes, Andrew Garbutt, Hollis Lau, Simon Letarte, Lukas N Mueller, Vagisha Sharma, Olga Vitek, Ning Zhang, Ruedi Aebersold, Julian D Watts

**Affiliations:** 1 Institute for Systems Biology, 1441 North 34th Street, Seattle, WA 98103, USA; 2 Institute of Molecular Systems Biology, ETH Zurich, Zurich, Switzerland; 3 Competence Center for Systems Physiology and Metabolic Disease, ETH Zurich, Zurich, Switzerland; 4 Department of Statistics and Department of Computer Science, Purdue University, West Lafayette, IN, USA; 5 Faculty of Science, University of Zurich, Zurich, Switzerland

## Abstract

**Background:**

Quantitative proteomics holds great promise for identifying proteins that are differentially abundant between populations representing different physiological or disease states. A range of computational tools is now available for both isotopically labeled and label-free liquid chromatography mass spectrometry (LC-MS) based quantitative proteomics. However, they are generally not comparable to each other in terms of functionality, user interfaces, information input/output, and do not readily facilitate appropriate statistical data analysis. These limitations, along with the array of choices, present a daunting prospect for biologists, and other researchers not trained in bioinformatics, who wish to use LC-MS-based quantitative proteomics.

**Results:**

We have developed Corra, a computational framework and tools for discovery-based LC-MS proteomics. Corra extends and adapts existing algorithms used for LC-MS-based proteomics, and statistical algorithms, originally developed for microarray data analyses, appropriate for LC-MS data analysis. Corra also adapts software engineering technologies (e.g. Google Web Toolkit, distributed processing) so that computationally intense data processing and statistical analyses can run on a remote server, while the user controls and manages the process from their own computer via a simple web interface. Corra also allows the user to output significantly differentially abundant LC-MS-detected peptide features in a form compatible with subsequent sequence identification via tandem mass spectrometry (MS/MS). We present two case studies to illustrate the application of Corra to commonly performed LC-MS-based biological workflows: a pilot biomarker discovery study of glycoproteins isolated from human plasma samples relevant to type 2 diabetes, and a study in yeast to identify *in vivo *targets of the protein kinase Ark1 via phosphopeptide profiling.

**Conclusion:**

The Corra computational framework leverages computational innovation to enable biologists or other researchers to process, analyze and visualize LC-MS data with what would otherwise be a complex and not user-friendly suite of tools. Corra enables appropriate statistical analyses, with controlled false-discovery rates, ultimately to inform subsequent targeted identification of differentially abundant peptides by MS/MS. For the user not trained in bioinformatics, Corra represents a complete, customizable, free and open source computational platform enabling LC-MS-based proteomic workflows, and as such, addresses an unmet need in the LC-MS proteomics field.

## Background

One area of particular interest to the proteomics community is the application of proteomics to the determination of proteins that are differentially expressed or abundant between samples representing different physiological or disease states [1-3]. Typically, such analyses require a quantitative proteomics approach, for which there is a wide range of experimental options available to the researcher. These generally fall into one of two categories, or represent some combined form of both [4]: i) stable isotope labeling, combined with LC-MS/MS identification, providing accurate relative abundance, or, if suitably calibrated peptide or protein reference samples are available, absolute quantification; ii) LC-MS label free quantification (i.e. pattern-based), in which quantification is determined via observed changes in the ion current for individual analytes.

Isotopic labeling and label free approaches each have their own set of challenges and limitations. MS/MS-based isotopic labeling approaches must introduce the label pre- or post-sample isolation. Post-isolation methods include the use of labeling reagents such as ICAT [5] and iTRAQ [6], whereas pre-isolation labeling methods (i.e. *in vivo*) include the use of SILAC [7] labeling reagents, for use in cell culture-based experiments. All of these methods, however, limit the number of individual biological samples that can be compared in a single experiment to a very low number, and peptides can generally only be quantified if they are also successfully identified by MS/MS, unless combined with a LC-MS profiling approach. In contrast, LC-MS-based label free approaches are ideal for the comparison of large sets of samples or populations where, in principle, every feature detected by the mass spectrometer is potentially quantifiable. However, since LC-MS approaches rely on some form of data alignment or pattern matching, they require a much higher degree of experimental reproducibility. This can be challenging for LC-MS, when large numbers of consecutive analyses are often required.

As a result of both the increased use of LC-MS-based workflows, and the complex computational challenge that the alignment of large sets of LC-MS data represent, a wide range of tools to address this need have appeared [8-16]. It is also because of the complexity of this problem, and the different computational approaches that can be taken to solving it, that each of the tools has its own, individual set of strengths and weaknesses. Thus for the biologist or proteomics researcher, tool selection can depend on what experiment is being done, or what mass spectrometer or data type is being used etc. For example, the two LC-MS tools we have implemented in the version of Corra presented here, SpecArray [12] and SuperHirn [15], each work better than the other with data generated with different types of mass spectrometer, as is discussed further below. Other tool limitations/considerations include: those designed to run on a single processor *versus *those that can run on a cluster; some have a limitation on the number of samples (data files) that can be aligned in one experiment; others are designed to use MS/MS identification of some peptides as 'landmarks' during the alignment process. In general, these LC-MS tools also have no, or only very minimal statistical capability to assign confidence to data alignments, and thus control the false discovery rate. Finally, as each tool was developed in a different academic setting, each with their own set of needs and workflows, the input/output formats of the tools do not make them readily compatible with each other, or the array of statistical packages that have been developed for analysis of high-dimensional data. For all these reasons, embarking on an LC-MS-based proteomic discovery workflow can be a daunting task for the biologist or other researcher who is not already well versed in proteomics and bioinformatics.

We therefore set out to develop, and here present Corra: a free, open source and customizable computational platform that enables LC-MS proteomic workflows. The Corra framework extends and adapts existing algorithms used for LC-MS-based proteomics, as well as existing statistical algorithms from the microarray community suitable for the analysis of high-dimensional LC-MS data, as well as adapting additional software engineering technologies, such as distributed processing and Google Web Toolkit. As such, Corra addresses an unmet need in the LC-MS proteomics field: to provide an open source computational platform that allows biologists and other researchers, not formally trained in bioinformatics, to easily process, visualize and analyze LC-MS data in the manner of their choosing, all within a single application, and on their own workstation. Using a web-based interface, Corra guides the user from MS data generation, through data processing, visualization, and statistical analysis steps, providing for multiple server-side data processing modes and statistical analyses along the way, towards the identification of differentially abundant or expressed candidate features for prioritized targeted identification by subsequent MS/MS. Corra also provides all the information and documentation required for the knowledgeable user to customize the platform, and integrate the data processing and/or statistical analysis tools of their own choosing, according to their specific workflow needs.

To illustrate the implementation of Corra for the analysis, visualization and interpretation of biologically relevant LC-MS data, we present analyses of two biological pilot studies as examples of commonly performed proteomic LC-MS experiments, that each highlight different aspects and uses of Corra. The first pilot study demonstrates the use of Corra for candidate plasma biomarker discovery aimed at human type 2 diabetes. Here we show the use of aligned LC-MS features to correctly classify the normal and disease plasma sample groups, followed by Corra-enabled targeted MS/MS identification of the differentially abundant peptide features. The second pilot study demonstrates the use of Corra for phosphopeptide profiling, where we identify *in vivo *substrates of a protein kinase using a kinase deletion strain of yeast. Here we expect that the target phosphopeptides of the kinase will be absent from one set of samples. Again, follow-up Corra-enabled targeted MS/MS identified the phosphopeptide features absent in the kinase knockout strain.

## Methods

### Overview of the APML format

To facilitate the integration into a coherent analysis platform of existing software tools with those yet to be developed, it was necessary to define a data format that could capture any and all relevant information relating to the data and the experiment, to ensure portability of data between the disparate tools in use. We therefore defined and implemented the Annotated Putative peptide Markup Language (APML) data format within the Corra framework to both store processed data, and port it from one tool to the next. APML is defined using eXtensible Markup Language (XML). We chose XML since it is a simple language, designed for data representation, and is easily parsed and self-describing by markup tags. APML captures essential LC-MS data information for statistical analysis, as well as additional information for identification, profiling, clustered features, etc. The APML schema and documentation can be found here [17].

**Figure 1:**
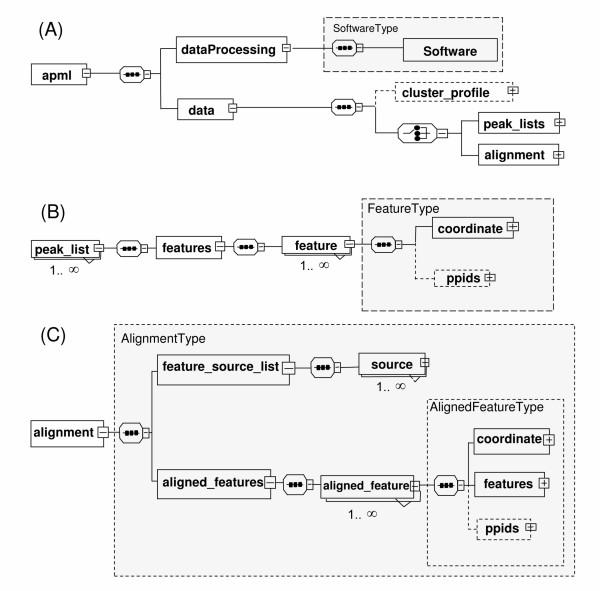
**Top elements of APML**. In the presented XML schema graph notation, dotted rectangles represent optional elements and solid rectangles represent required elements. Complex types, which can be used as common element types, are defined by shaded boxes. Elements with "+" indicate there are further subelements and elements with "-" indicate that it has been expanded to display in the figure.  indicates sequence type of child elements and  indicates choice type of child elements. A) The apml element has two child elements. The dataProcessing element stores software information, and data element child elements of either feature list as peak_list element, or alignment feature list as alignment element. The cluster_profile element is an optional element for a list of clustered feature references in any time course or dilution series experiment. The dataProcessing element stores software information, and data element stores either feature list as peak_list element or alignment feature list as alignment element. B) The peak_lists can have one to many peak_list elements, which stores the detected features of a single LC-MS run. C) The alignment element stores all LC-MS file information in feature_source_list, and aligned features are stored in aligned_features element.

The apml element has two child elements, the dataProcessing and data elements (Figure 1A). The dataProcessing element only stores software information in the SoftwareType, while the data element, and all its sub-elements, stores all data information and potential annotations. The primary elements for data storage are the peak_lists and alignment elements. However, there is also an optional cluster_profile element, which, for example, can be used to capture a list of clustered feature references, such as would be found in a time course or dilution series experiment, when needed. Picked feature lists are stored in peak_lists, and can have one to many peak_list elements, where each stores the detected features of a single LC-MS run (Figure 1B). The alignment element stores all the LC-MS file information in the feature_source_list element, and stores aligned features in the aligned_features element (Figure 1C).

**Figure 2:**
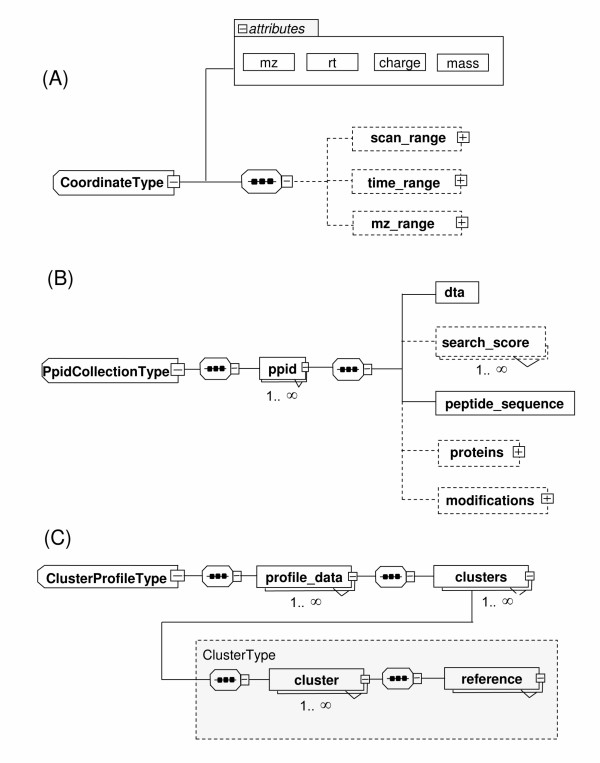
**Additional elements of APML**. XML schema graph notation is the same as described in Figure 1 above. A) Both FeatureType in peak_list and AlignedFeatureType in alignment elements have CoordinateType, which contains coordinates for each feature, defined by required attributes of *m/z*, rt (retention time), charge and mass. It also has optional retention time, *m/z *and scan range child elements. B) We also defined the optional PpidCollectionType element for each feature, to store putative feature identification, via MS/MS tandem mass spectrometry experiments and/or other existing database references. C) ClusterProfile Type element is to store grouped features, by referencing the features defined in either peak_list or alignment, since some post-alignment processing tools might need to cluster LC-MS processed features by some criteria. For example, features whose intensities display a correlation with a sample concentration dilution series can be grouped and stored in this optional element.

Both the FeatureType and AlignedFeatureType elements (shaded boxes, Figure 1B and 1C) have the coordinate element. The coordinate element is defined as CoordinateType (Figure 2A), which contains coordinate information defined by *m/z *(mz), retention time (rt), charge and mass attributes. The CoordinateType also contains optional range elements for scan, time and *m/z*. We also defined PpidCollectionType for the ppid element for each feature (Figure 2B). This is an optional element to store putative feature identification that can be derived from MS/MS data, or via existing database information, such as from PeptideAltas [18] or UniPep [19]. Finally, we also found it useful to have an element, the optional ClusterProfileType (Figure 2C), to store a grouped collection of any type of intensity profile that may arise through a sample dilution series or time course, for example. Full descriptions of all elements of APML and its types can be found in the APML documentation [17].

### APML implementation with LC-MS and statistical tools

A goal of Corra was to enable the integration of multiple and disparate LC-MS data analysis tools, and integrate them, seamlessly, with common statistical packages to allow for better comparison between differently-processed datasets, via the addition of statistical measures of confidence and error rates. The integration of tools was achieved via AMPL and the various parsers. In the current build of Corra we have integrated SpecArray [12] and SuperHirn [15], both adapted to generate their final output in APML, as well as to operate in a distributed computing environment. To integrate the tools with the needed statistical analyses, we implemented another APML parser, using R's XML library, to facilitate an interface to CorraStatistics.R. This allows the user to launch within Corra additional statistical data analyses, suitable for quantitative proteomics data analysis, via the use of selected Bioconductor packages.

Bioconductor is a leading open source and software project for the statistical analysis of high-throughput biological data [20], and is primarily based on R, a language and environment for statistical computing and graphics. Bioconductor represents a rich source of statistical packages pertinent to proteomic data analysis, and thus a perfect source of tools for Corra's data processing framework. The current version of CorraStatistics.R includes the LIMMA [21] and maSigPro [22] Bioconductor packages to detect statistically significant features that are differentially abundant between related sample sets (e.g. disease control *vs*. case) and with a controlled false discovery rate, with the option of adding time course information, if required. CorraStatistics.R also allows for the use of MLInterfaces and Hierarchical Clustering for supervised and unsupervised clustering applications.

An inherent aspect of any LC-MS profiling experiment, where features are to be aligned across multiple LC-MS runs, is the issue of missing features across a subset of the LC-MS runs being aligned. One explanation for such 'missing' features is the failure of the feature detection and/or alignment tools to correctly detect and align every true feature in every data file, usually as a result of experimental variation (e.g. variations in LC retention, signal to noise, *m/z *drift, etc.). However, missing features may also be indicative of real and desired experimental information. Therefore, instead of throwing away aligned features with missing intensity values of a subset of the LC-MS runs, CorraStatistics.R also provides the user with the option to replace such missing intensity values, either with a user-specified value, or the default setting: the minimum intensity value obtained among all aligned features, after intensity normalization, for the given data set. These missing values are then applied to the aligned intensity dataset, allowing subsequent analyses to detect features that were above the limit of detection by LC-MS in one data/sample sub-group, but not in another.

### APML Parsers and viewers

**Figure 3:**
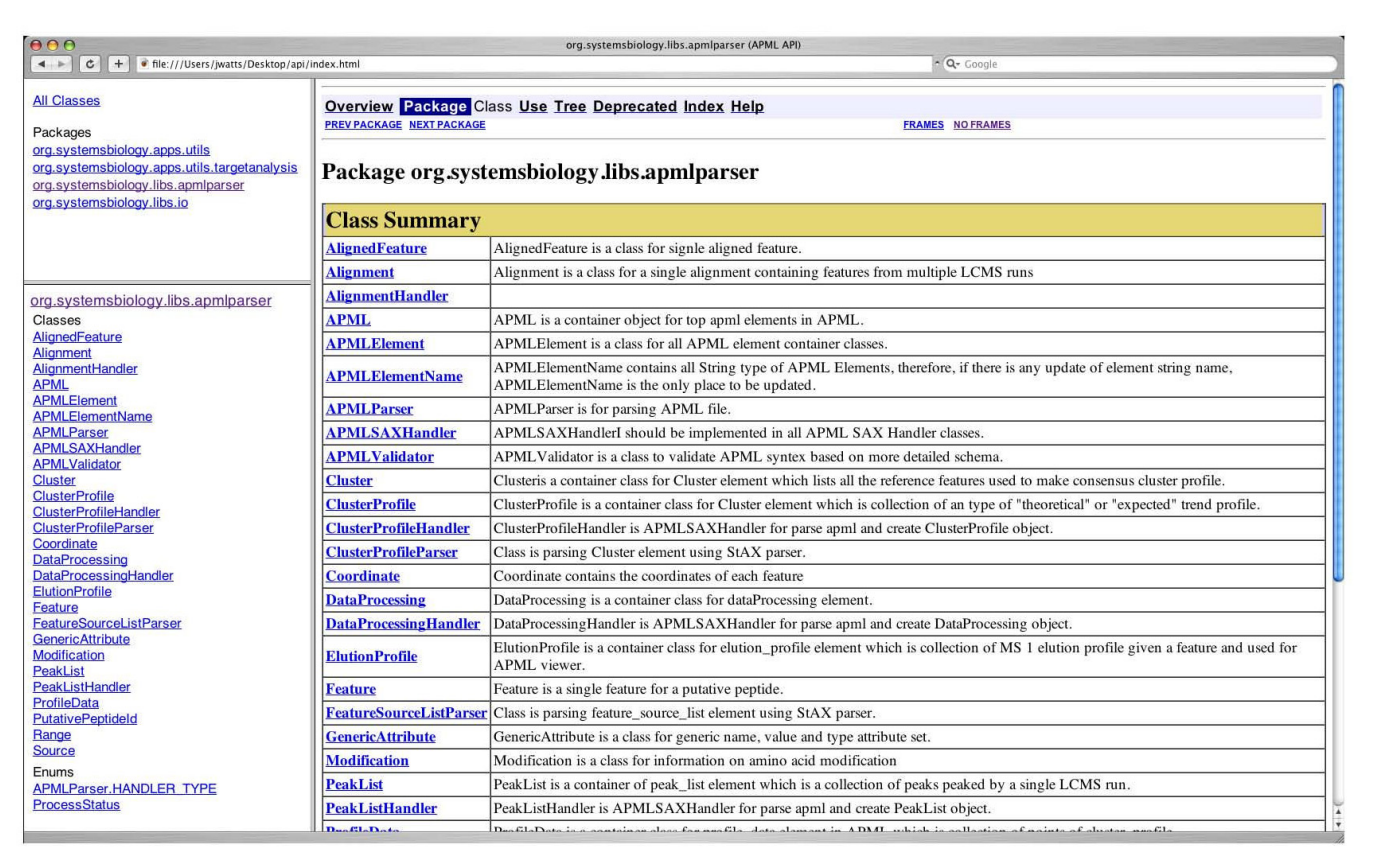
**APML parser documentation**. Corra software provides an APML parser package written in java. This is to facilitate Corra customization via the adaptation of existing software or analytical components, or importing of new software or analytical components, as required by users with specific workflow needs. This figure shows an example screenshot of the parser package documentation.

**Figure 4:**
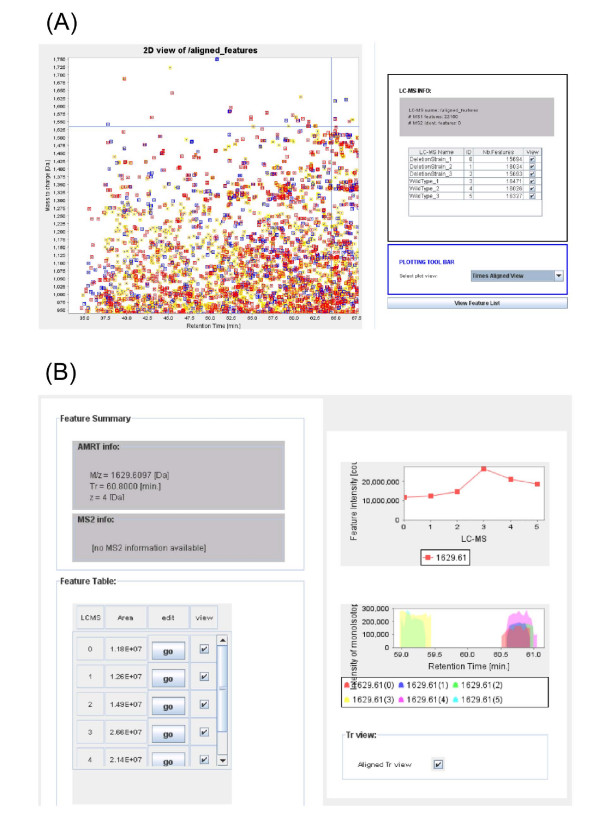
**APML viewer**. Corra also provides a 2D graphical APML viewer, for user-friendly visualization of peak list or aligned APML files. A) It allows for color-coding of displayed features according to observed charge state, or the number of LC-MS runs the feature was successfully aligned for. Feature coordinates can be zoomed in and out to allow viewing of entire APML files, or just regions of particular interest. B) When a given aligned feature is selected, a pop-up window will be displayed for that feature across all LC-MS runs in the dataset.

To enable Corra to launch multiple and disparate tools, as well as to facilitate the integration of other new and yet to be developed LC-MS tools into Corra, we needed to implement APML parsers and enable the use of APML across the whole Corra platform. We thus implemented a generic APML parser library package using Java Standard Edition 6. To ensure efficient memory and parser use, Simple API for XML and Stream API for XML were used in the library package. The APML parser package is also included with Corra, along with the APML schema documentation, to the quantitative proteomics tool development community, to enable the integration of other pre-existing and newly developed tools into the Corra framework, and thus enable other workflows and applications of Corra on an 'as needed' basis. The APML schema and implementation are also readily extensible, and thus customizable, via the addition of new optional elements and by extending the provided java classes. The Javadoc, org.systemsbiology.libs.apmlparser, is thus provided to assist any developer who wishes to plug the current parser into another analytical tool implementation, for his or her own specific needs (see Figure 3). APML peaklist and alignment viewers are also implemented within Corra to provide the user with a visual 2D display of LC-MS intensity data, within *m/z *and LC retention time dimensions (see Figure 4).

### Corra web graphical interface

Another important design goal for Corra was to enable relative ease of use of its various tools and statistical packages by users with little or no formal bioinformatic training, all within a single computational interface. This was accomplished via the development a web-based graphical user interface (GUI) to help guide any user through the various desired data processing and analysis steps, in a systematic and straightforward way. This was done by using Google Web Toolkit for the client web application. Access to the interface is via a web-browser, and does not require installation of any custom software on the user's computer. Data processing happens on a remote server that leverages a compute cluster environment to achieve high-throughput and scalability. This has the advantage of not tying up the user's computational resources during time-consuming analyses.

**Figure 5:**
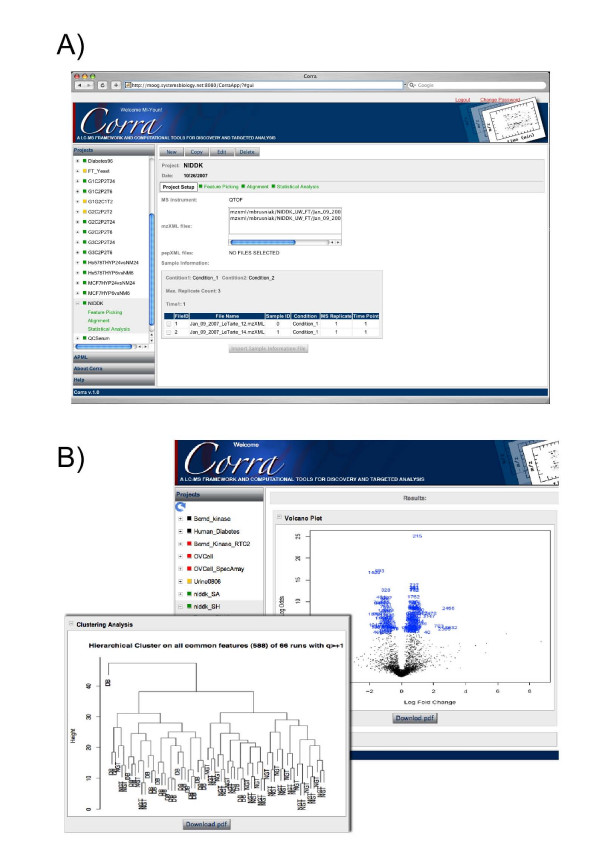
**Corra graphical user interface (GUI)**. Example screenshots of the Corra GUI, provided as a web client using Google Web Toolkit. The GUI guides users, step by step, through the Corra pipeline, and also to serves to organize data by project, in a user-friendly way, not requiring extensive knowledge of computational biology. A) Project setup GUI panel guides project organization and status. B) Analysis GUI panel displays figures from analyses.

Figure 5A shows the Corra project setup page, where the user can create new, as well as retrieve existing projects. In addition to guiding the user through the Corra workflow, the graphical interface also allows the user to monitor the processing status of a project, as well as visualize the analysis results when they are available. The Project setup page also captures meta-data information, which can also be used during statistical analysis. The user must indicate "Sample ID" (same as individual ID), "Condition", "MS Replicate" and "Time Point" information for each LC-MS run (more than two conditions can be used for a statistical contrast study). Figure 5B shows the analysis panel, in which users can view APML outputs in a plotted graphical format, or outputs from CorraStatistics.R. A tab delimited file, which can be used as an inclusion list for follow-up targeted MS/MS analyses, is also available for download to the client computer.

In addition to the number of LC-MS runs to be analyzed and their inherent file-sizes, the processing time is highly variable and dependent on which LC-MS tool is used, user specified setup parameters, as well as the hardware and number of compute nodes that the Corra platform is run on. However, as a guide, utilizing SpecArray for alignment in a multi-threaded and distributed computing environment, feature extraction for 105 LC-MS runs, with a ~3 GB mzXML file per LC-MS run, took Corra ~25 hours. This was done on a six dual-core, dual processor AMD Opteron 275 (2.2 GHz) cluster, each with 1 MB of level 2 cache.

### Implementing new LC-MS tools or statistical packages in Corra

As has been discussed above, the version of Corra presented here includes implementation of SpecArray [12] and SuperHirn [15] for the LC-MS data alignments, and the LIMMA [21] and maSigPro [22] packages for statistical analysis of aligned data. However, researchers with a sufficient bioinformatics background can readily implant additional tools of their choosing. To add an additional LC-MS tool, this would minimally require modifying the new tool's code to be able to input and output data in APML format. Tools that also use MS/MS data would have to be modified to use pepXML [23] representation of the MS/MS data. Additionally, tools that are not designed to do so could be modified to take advantage of Corra's distributed processing architecture, if desired. Since Corra already includes parsers for R's ExpressionSet data format, introducing existing or new R statistical packages is very simple. Finally, the Corra GUI would have to be updated to show the new tools in the web interface.

### Enrichment of *N*-glycopeptides from human plasma samples relevant to type 2 diabetes

22 human plasma samples were obtained directly from the funding agency (i.e. from the NIDDK, collected specifically for the project PAR-04-076 [24]). Diagnostic classifications had been made via the oral glucose tolerance test (OGTT), the current diagnostic 'gold standard' for diabetes. 13 control samples were from subjects with normal glucose tolerance (NGT), with blood glucose levels ranging from 54 to 98 mg/dl 2 hours post-glucose challenge after fasting. 9 samples were from patients newly diagnosed with type 2 (adult onset) diabetes (DB) (i.e. had not received treatment, or made lifestyle adjustments), with blood glucose levels ranging from 202 to 279 mg/dl 2 hours post-glucose challenge after fasting. *N-*linked glycoproteins were enriched from the plasma samples, and the formerly *N-*glycosylated peptides recovered, following digestion with trypsin and protein *N*-glycosidase F (PNGase F) sequentially, essentially as described elsewhere [25]. Sample preparations were performed in a 96-well plate format, using a Freedom Evo robotic workstation (TECAN, Maennedorf, Switzerland) for all liquid handling and incubation steps, following manual transfer of plasma samples to the 96-well plate. Sample locations in the plate were randomized and recorded. Final *N-*glycopeptide isolates were stored, dry, in glass vials at -80°C until needed. Samples were resuspended in 0.4% acetic acid prior to MS analyses.

### Mass spectrometric analyses of human plasma *N*-glycopeptide isolates

Peptides were separated on an 1100 Series HPLC system (Agilent, Santa Clara, CA) equipped with a nanoflow pump, operating at a flow rate of 1 μl/min. Mobile phase A was a 0.1% formic acid in water, and mobile phase B was 0.1% formic acid, 5% water, 95% acetonitrile. A binary linear gradient from 5% to 35% B was used to separate the peptides on a 10 cm monolithic C_18 _column, with a 100 μm inner diameter (Merck KGaA, Darmstadt, Germany). A self-packed integraFrit column (new Objective, Woburn, MA) with a bed of magic C_18 _5 μm particles (Michrom Bioresources, Auburn, CA), 2 cm × 100 μm, was used as a pre-column. Sample volumes of 5 μl were injected by the autosampler. Samples were randomized for loading, and re-randomized for all subsequent technical replicates.

Mass analysis was performed on a micrOTOF electrospray time-of-flight mass spectrometer (Bruker Daltonics, Billerica, MA) at a mass accuracy of 5 ppm, and a resolving power of 9,000 or better. The mass scale was calibrated using glu1-fibrinopeptide B (Glufib)(Sigma, Saint-Louis, MO) and mass spectra were acquired at 1 spectrum/s over an *m/z *range of 300–1,600. High mass on the micrOTOF was maintained via automated instrument recalibration between each sample injection. This was achieved by injecting 320 fmol of Glufib, before running a 15 minute wash gradient, increasing the cone voltage to 220 V to induce in-source CID. The Glufib fragment ions were then used by a visual basic script to recalibrate the mass spectrometer on-the-fly, thereby ensuring high mass accuracy from the first to last sample. This measure also has the benefit of greatly reducing carry-over between complex samples, such as plasma-derived peptide isolates, and also provides a way to monitor sensitivity and reproducibility of the system during large-scale sample batch analyses.

For MS/MS identification of Corra-identified discriminatory *N-*glycopeptides, an inclusion list for the top-ranked 400 discriminatory peptides was used for targeted MS/MS on an LTQ-FT mass spectrometer (ThermoFisher, San Jose, CA) as described elsewhere [26].

### Enrichment of phosphopeptides from control and Ark1 kinase knockout strains of yeast

Three biological replicates each of the yeast *S. cerevisiae *wild type (BY7092: can1::STE2pr-Sp_his5 lyp1Delta his3Delta leu2Delta ura3Delta met15Delta) and the deletion strain (BY7092: can1::STE2pr-Sp_his5 lyp1Delta his3Delta leu2Delta ura3Delta met15Delta ark1Delta) were grown to OD ~0.8 at 30°C in synthetic defined (SD) medium (per liter: 1.7 g YNB, 5 g ammonium sulfate, 2% glucose (w/v), 0.03 g isoleucine, 0.15 g valine, 0.04 g adenine, 0.02 g arginine, 0.1 g leucine, 0.03 g lysine, 0.02 g methionine, 0.05 g phenylalanine, 0.2 g threonine, 0.04 g, 0.04 g tryptophan, 0.03 g tyrosine, 0.02 g uracil, 0.1 g glutamic acid and 0.1 g aspartic acid). Cells were harvested at 30°C by centrifugation. Then cells were washed once in SD medium (at 30°C), pelleted by centrifugation, and were shock-frozen in liquid nitrogen until needed. Yeast cell pellets were subsequently thawed in ice-cold lysis buffer (20 mM TrisHCl pH 8.0, 100 mM KCl, 5 mM EDTA, 20 nM calyculin A, 200 nM okadaic acid, 4.8 μm cypermethrin (all from Merck KGaA, Darmstadt, Germany), 2 mM vanadate, 10 mM sodium pyrophosphate and 10 mM NaF) using 1 ml of lysis buffer per gram of yeast. Yeast cells were lysed by glass bead beating (using acid washed glass beads). Phosphopeptides were isolated following tryptic digestion of total protein isolates using titanium dioxide affinity purification, as described elsewhere [27,28].

### Mass spectrometric analyses of yeast phosphopeptide isolates

The phosphopeptide samples were analyzed on a hybrid LTQ-OrbiTrap mass spectrometer (ThermoFisher Scientific, Bremen, Germany) interfaced with a nanoelectrospray ion source. Chromatographic separation of peptides was achieved on an Eksigent nano LC system (Eksigent Technologies, Dublin, CA, USA), equipped with a 11 cm fused silica emitter, 75 μm inner diameter (BGB Analytik, Böckten, Switzerland), packed in-house with a Magic C_18 _AQ, 5 μm beads, loaded from a cooled (4°C) Spark Holland autosampler, and were separated using acetonitrile/water solvent system containing 0.1% formic acid, at a flow rate of 200 nl/min. Peptide mixtures were separated with a gradient from 5 to 30% acetonitrile over 80 minutes. For MS/MS data acquisition, one data-dependent MS/MS scan was acquired in the linear ion trap for each OrbiTrap-MS scan, the latter acquired at 60,000 nominal resolution settings (full width at half maximum), with an overall cycle time of ~2 seconds. Charge state screening was employed to select for 2+ ions, rejecting 1+ ions and those with undetermined charge. For injection control, the automatic gain control was set to 5 × 10^5 ^and 1 × 10^4 ^for full OrbiTrap-MS and linear ion trap MS/MS, respectively. The instrument was calibrated externally, according to manufacturer's instructions. Data was acquired using internal lock mass calibration on *m/z *429.088735 and 445.120025.

### Database searching of MS/MS data for peptide identification

Data from targeted LC-MS/MS on an LTQ-FT, for the human plasma study, were searched using SEQUEST v27, and the human IPI database v3.23 (which contains 66,617 proteins). Trypsin was specified for cleavage, allowing one non-tryptic terminus. No mass filtering was used, a 0.1 Da precursor mass tolerance was used, and a fragment ion tolerance zero was applied (which in this version of SEQUEST corresponds to a tolerance of ~0.5 Da due to the unit mass binning that SEQUEST applies to the input spectra). A stable modification for Cys of +57.05 Da was used, as well as differential modifications for Met of +16.0 Da and for Asn of +0.984 Da. A maximum of 4 modified residues per peptide were allowed. OrbiTrap MS/MS data, for the yeast kinase study, were searched using SORCER ER-SEQUEST v3.0.3, running on SageN Sorcerer, and using the Yeast SGD database (Version of 10.20.2007, which contains 6,795 forward protein entries and 6,795 reversed protein entries). Trypsin was specified for cleavage, allowing for two missed cleavages and one non-tryptic terminus. Mass tolerance was set to 25 ppm for the monoisotopic precursor ions, and to 0.5 Da for fragment ions. Stable modification for Cys of +57.0214 Da was used, along with stable modification for all carboxylate groups of +14.0156 Da, and differential modification for Ser, Thr and Tyr of +79.9663 Da was also used. For both datasets, final peptide assignments were made and false discovery rates calculated by PeptideProphet (v3.0) interpretation of SEQUEST search results [29]. For the purposes of this study, modified amino acid assignments, according to above criteria, were made by virtue of the top-ranked SEQUEST match, followed by manual/visual inspection of MS/MS spectra.

## Results and discussion

**Figure 6:**
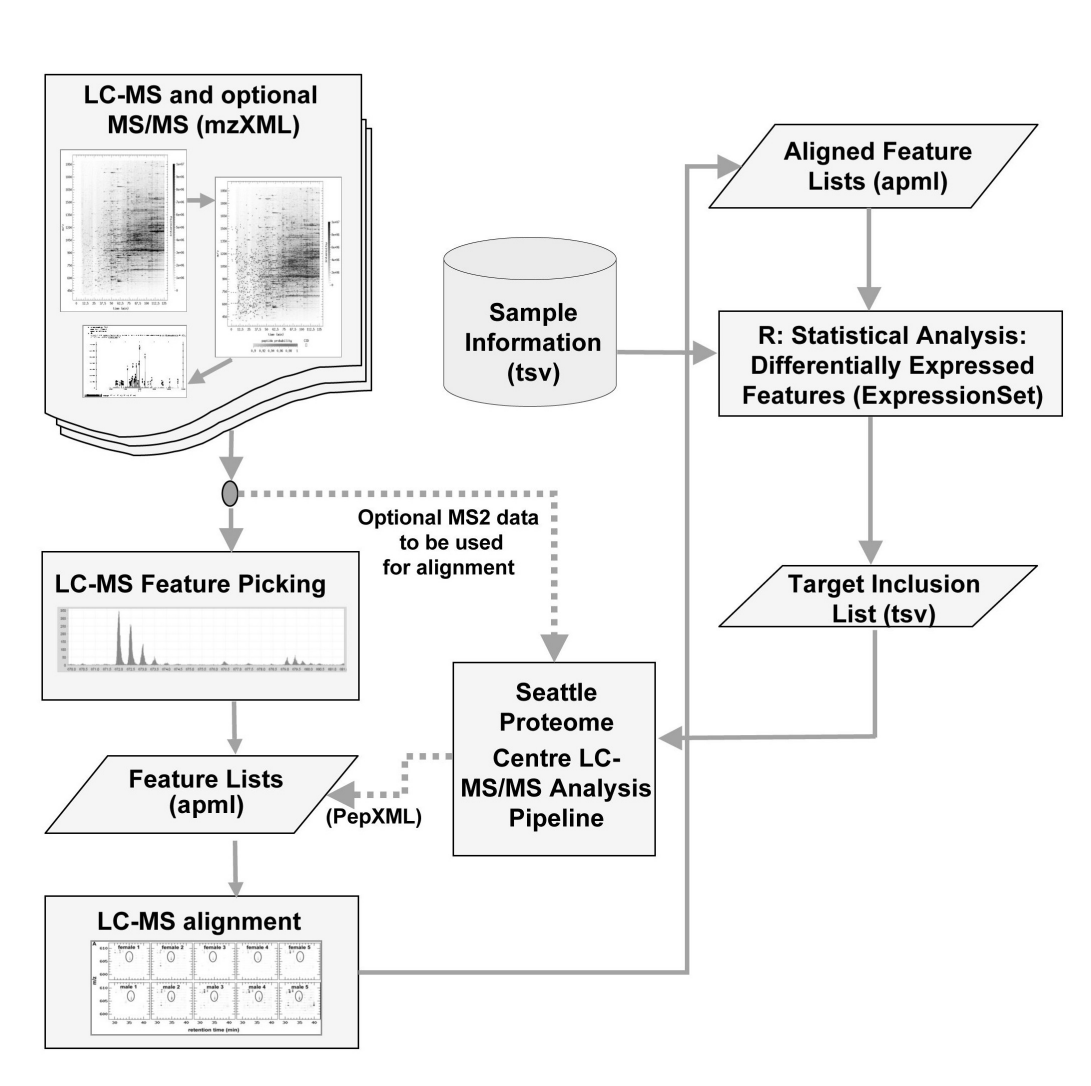
**Summary of the Corra framework data flow**. In the flow chart, the rectangular boxes represent one or more software processing steps, parallelograms represent data, and the cylinder represents databases. The application of Corra begins with the input of data in mzXML format, converted from the raw files from any of various mass spectrometers capable of producing sufficient resolution to resolve isotopic distribution. Features (defined by *m/z*, retention time, and intensity) for each input LC-MS run are extracted, based on observed isotopic distribution, and with the resultant peak list stored in APML format. Extracted features are then aligned across all LC-MS runs for the dataset in question, with the resultant aligned features list also and stored in the aligned APML format. The xml format of the aligned APML is then parsed into standard R data format, ExpressionSet, prior to statistical analyses. Statistical tests, using linear mixed model, are performed on all the aligned features, together with any relevant biological and technical replicate information in the sample set. The current implementation of Corra has adapted the previously published LC-MS quantification software tools SpecArray [12] and SuperHirn [15] for feature extraction and alignment.

As has been previously stated, the goal of the Corra project was to create a single computational platform that was customizable, free and open source, for the enabling LC-MS-based proteomic workflows. The sections that follow below serve to illustrate the data flow through the Corra framework, and include discussions of the processing/analysis options available in the Corra implementation presented here, summarized in Figure 6. This is then followed by sections describing two, very different, biological pilot studies, chosen as 'real world' experimental examples, both to illustrate and validate the application of key aspects of the Corra workflow to quantitative LC-MS data processing and analysis, and it's use for informing subsequent identification of peptides/proteins of interest via targeted MS/MS.

### Corra data input and software processing

At the outset, (multiple) raw LC-MS data files are first converted to mzXML [30], prior to importing into the Corra framework. The data can then be processed for feature/peak picking and alignment. The current implementation of Corra uses SuperHirn for very high mass accuracy OrbiTrap or FT-MS data (and is the default tool setting for such data, unless otherwise specified by the user) and SpecArray for high mass accuracy TOF-MS data (similarly the default tool setting for these data). These default settings were, in fact, determined through testing the feature picking/alignment tools on multiple data types, where we observed that a given tool performed better with certain types of MS data, and in a somewhat instrument-dependent way. We reasoned that this effect likely resulted from the original data sets that were used during the initial stages of tool development and testing. SpecArray was initially developed for the analysis of ESI-TOF data [12], and thus performed better than SuperHirn for the analysis of the ESI-TOF data, such as that obtained for the human type 2 diabetes plasma study shown below. On the other hand, SuperHirn was initially developed for the analysis of very high mass accuracy FT-MS data [15], and thus performed better than SpecArray for the analysis of very high mass accuracy OrbiTrap FT-MS data, such as that in the yeast kinase knockout study, also shown below. Since it is necessary to have high mass accuracy data (i.e. from TOF or FT mass spectrometry platforms) in order to perform LC-MS profiling of complex samples, the implementation of SpecArray and SuperHirn in this initial version of Corra represents sufficient choice for anyone wanting to perform LC-MS profiling using Corra. However, the Corra platform, being open source, was designed so that additional tools could be integrated, according to project-specific needs, as described under Methods above.

Another feature of the Corra framework is that it facilitates the process of peak picking and alignment on the server side (thus not tying up the user's own computer) utilizing an underlying cluster environment with a job-scheduling system (in this case a Portable Batch System) to process all the LC-MS runs in a given data set. This mode of analysis also ensures that the process is not limited by the size of the data set (i.e. the number of LC-MS runs) as some stand-alone tools can be. Following feature detection, the user can display pertinent statistics to evaluate the data quality and usefulness, such as a distribution for the number of features extracted from each LC-MS run. Finally, the outputs of these analyses are converted to APML, both for storage, and for data visualization via Corra's APML viewer (see Figure 4 and above under Methods).

### Corra peak picking and alignment

As discussed above, the current implementation of the Corra framework allows for the use of either SpecArray or SuperHirn for feature picking and alignment purposes. However, in order to facilitate the analysis of large data sets, where MS signal intensity typically varies over time, Corra normalizes the MS signal intensity data, prior to importing into the peak picking and alignment tools, using LIMMA (Linear Models for Microarray Data) [21]. Corra, via APML, also allows for subsequent annotation of aligned peaks where MS/MS data is available, for example via subsequent targeted MS/MS identification of differentially abundant peptide features.

It is worth noting here that Corra was designed primarily for the analysis of LC-MS-based (i.e. label free) quantitative proteomic data. It is thus highly desirable that the data itself should be acquired under conditions that maximize for reproducibility. To this end, in one of the studies discussed below, we instituted an automated calibration of the mass spectrometer via the inclusion of a calibration standard in the blank/wash cycle. This provided for very consistent mass resolution and accuracy, meaning that the major concerns to focus efforts on were the maintaining reproducibility for both chromatography and sensitivity. If sensitivity were to drop appreciably, then many features that were above signal-to-noise may no longer be detected. This effect could mislead the user into thinking that the absence of such features was related to biology, rather than machine performance. It is therefore important to be mindful of this issue when analyzing large LC-MS profiling datasets. Indeed, the use of the calibration standard between runs enabled us to closely follow MS sensitivity over the course of large-scale experiments, and several datasets were abandoned before we obtained the data presented in the diabetes example study below, due to the detection of MS sensitivity-related problems. Reproducibility of LC retention time, on the other hand, is somewhat more challenging, the main issues being sample carry-over and gradient drift. Fortunately, the short wash cycle between analyses greatly reduced carry-over, without significantly increasing the time required per sample analysis. Gradient drift can be harder to control. However, improvement in the alignment algorithms currently implemented, have meant that, for the higher-end LC systems commonly in use, this is rarely an issue, save for a major breakdown in the LC-system.

### Statistical data analysis using CorraStatistics.R

To be complete, any single platform for LC-MS data analysis would need to include statistical algorithms, appropriate for the analysis of LC-MS data, to generate measures of significance for (peptide) features that appeared to be differentially expressed or abundant between two or more sample groups. Fortunately, many such algorithms have been established for the analysis of genomic and microarray data, now freely available via Bioconductor [20]. Corra thus includes Bioconductor R statistical packages that are useful and appropriate for the interpretation LC-MS data to meet this need, and which are contained in the CorraStatistics.R module.

To perform statistical analyses, APML data sets are first imported into CorraStatistics.R, which first parses APML's dataset and sample information to create an annotated sample and feature intensity data format in the ExpressionSet [31] object, the format required for application to the R's statistical packages from Bioconductor. The implementation of CorraStatistics.R presented here, uses LIMMA [21] for processing data without time course information, and maSigPro [22] for data that does contain time course information. Regardless of which is used, the final output is always the same: a ranked list of features that best discriminate between one or more biological/physiological/clinical groups. This list can then be used to generate an inclusion list for targeted MS/MS analysis and subsequent identification of the discriminatory peptides/proteins of interest (see Figure 6). Finally, the MS/MS spectra, and resultant peptide sequences identified etc., can be annotated back into the aligned APML file for that particular data set.

One drawback of clustering analyses, and indeed many other statistical methods that could be applied, is that they can only use features that aligned across all LC-MS runs. However, it is possible that a given feature may not be present in one sample pool *versus *another due to a genuine biological effect, rather than it being below the limit of MS detection in one or more LC-MS runs, or due to an error made by either the feature picking or alignment tool. Thus, in order to work around the clustering limitations for such real-life situations, we included an optional function within Corra (called 'n/a replace'), where the user can replace missing intensity data (i.e. given features not aligned across all LC-MS-runs) with the minimum measured intensity for the entire dataset (the default setting), or a specified nominal value of their choosing. When missing intensity values are not replaced, only features that have intensities across all LC-MS runs will be used for supervised or unsupervised clustering analyses. LIMMA analysis of aligned features can then be performed to calculate fold-changes in intensity for each aligned feature across all LC-MS runs which, in turn, can be used to assign a measure of statistical significance for the observed fold changes, for the given dataset. When missing values are replaced prior to clustering, this will produce highly artificial ratios, which can be very misleading if interpreted improperly. Therefore, great care must be taken in applying this optional functionality prior to clustering analysis. For example, if a given feature in the 'control' population aligned across 19 of 20 runs, then replacing the missing feature could be beneficial, since this is likely a 'real' feature that was missed in just one run by the MS or software tools. However, it may be unwise to replace all 17 missing features for another feature that aligned across only 3 runs.

Nevertheless, there are clearly situations where the ability to replace missing features with a nominal value are of use, hence the provision of this function. An example of such a case is given below, where LC-MS profiling was performed on phosphopeptides isolated from a specific protein kinase knockout strain of yeast, in comparison to a wild-type control strain. In this case, we expected missing features in the knockout, when compared with the control. Thus, by using the missing feature replacement function wisely, we were able to successfully cluster the data to identify phosphopeptides that were not present in the profile from the knockout yeast strain. Similarly, in a typical biomarker discovery workflow, there may be markers only present (or absent) in the disease samples, due to a change in gene expression *versus *the control samples. Thus if one were to observe features that aligned across all (or most) cases, but not the controls, the judicious use of this function would similarly help identify such features. However, since the ratios it generates are highly artificial and therefore open to misinterpretation, it is up to the individual user to ensure that they use this particular function wisely, and to report it when they do so. Indeed, it should be stressed that there are many ways in which high-dimensional data, such as LC-MS data, can be validly analyzed. Thus it is always incumbent on the individual user to first consult with the literature, and/or a suitably qualified biostatistician, before embarking on such complex statistical analyses. Finally, the Bioconductor packages implemented so far were chosen for applicability to our current proteomics research. However, alternative approaches to both statistical data analysis and missing feature replacement are enabled by Corra's open software architecture. With the built-in converter to R's ExpressionSet file format, a user can readily extend or plug-in their own Bioconductor packages of preference into the Corra pipeline, as discussed under Methods above.

### Examples of Corra application to biological studies

We next used Corra for the analysis of LC-MS data from two biological pilot studies, as examples of commonly performed proteomic LC-MS experiments. The first goal of these biological studies was to validate the Corra platform's capability to take LC-MS data all the way through to the identification of statistically credentialed, differentially abundant peptides for targeted MS/MS identification. The second goal was to provide 'real life' examples of discovery-based proteomic experiments to illustrate the type of experiments Corra is useful for analyzing, and to show the type of information it can provide for the biologist end user. These two studies were also chosen since they separately highlight different aspects of, and the flexibility of Corra.

The first of these is from a pilot type 2 diabetes biomarker discovery project using human plasma. Here we wanted to be able to initially classify the samples according to disease state via label-free LC-MS analysis, then subsequently identify differentially abundant peptides via MS/MS. The second is from a study to identify candidate protein kinase substrates *in vivo *via LC-MS phosphopeptide profiling, using kinase deletion strains of the yeast *S. cerevisiae*. Here we show an example using a yeast deletion strain for the kinase Ark1. In this case, unlike the type 2 diabetes study, we expected the phosphopeptides of interest to be completely absent from the LC-MS profiles compared with a wild-type strain, requiring a different analytical strategy using Corra. Again, we subsequently identified the missing phosphopeptides of interest by MS/MS reanalysis of the wild-type yeast strain.

### Application of Corra to plasma biomarker discovery for human type 2 diabetes

The purpose of the pilot study presented here was to apply current LC-MS quantitative proteomics technology to try and identify potential type 2 diabetes candidate plasma biomarkers via profiling of (formerly) *N-*glycosylated peptides (*N*-glycosite peptides). To do this, *N*-glycosite peptides were isolated from plasma samples collected from control individuals with normal glucose tolerance (NGT), as well as from newly diagnosed cases of type 2 diabetes (DB).

*N*-glycosite peptide isolates were thus prepared from 13 individual NGT plasma samples and 9 individual DB plasma samples, as previously described [25] and summarized above under Methods. The 22 samples were then randomized for LC-MS analysis, followed by 2 additional technical LC-MS replicate analyses of all 22, each with a new randomization of sample run order to reduce potential bias, for a total of 66 LC-MS runs. Following conversion of the raw data to mzXML format, the 66 data files were input into Corra for feature picking and alignment by SpecArray. The aligned datasets were then imported into the CorraStatistics.R module for statistical analysis, as described above.

**Figure 7:**
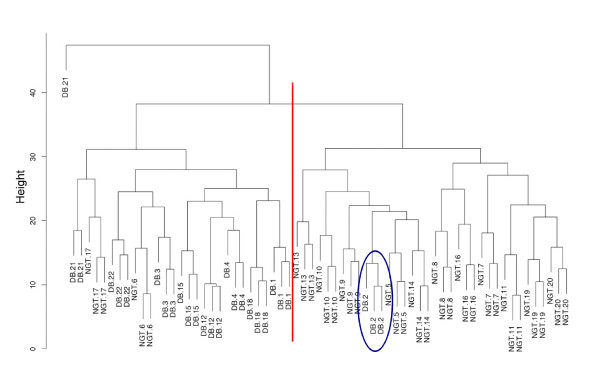
**Corra-generated hierarchical clustering of human type 2 diabetes plasma analyses**. *N*-glycosite peptides were isolated from human plasma samples and analyzed via LC-MS, as described under Methods. Randomized, triplicate analyses were performed for each of 22 human plasma samples, 13 controls (NGT: normal glucose tolerance) and 9 from newly diagnosed cases of type 2 diabetes (DB). The hierarchical cluster shown is for the 558 multi-charged features that aligned across all 66 LC-MS runs. Randomly assigned patient numbers are included to show how the replicate MS analyses of the same samples clustered together as the most similar, as expected. One of the misclassified DB patient samples was annotated as from a 'likely not fasted' subject, as required by the OGTT assay used to diagnose diabetes, according to documentation provided with the samples.

Figure 7 shows an example of a Corra analysis output, in this case the result of an unsupervised hierarchical clustering of the 66 LC-MS runs for the 13 NGT and 9 DB patients. For this particular analysis, the clustering algorithm utilized the 588 multiply charged features (i.e. excluding 1+ ions) that had been aligned across all 66 runs. This unsupervised cluster dendrogram showed good separation between the two diagnostic groups. It also showed that, as would be expected, the 3 replicate LC-MS runs of each sample were consistently the most similar to each other. The cluster also identified one particular outlier (indicated in Figure 7) that was explainable. The plasma sample from this particular individual was also annotated with a high blood triglyceride measurement. Thus this person had likely not fasted, as required, before the OGTT used to make the physiological state classification, and thus the results of the OGTT are not reliable. The cluster also shows that two additional NGT individuals were misclassified. However, this is more likely to be due to natural variation from one individual to the next when trying to diagnose a physiological state via LC-MS profiling alone, rather than due to the method of analysis. It is also worth noting that the OGTT itself, used to define the sample populations, while being the current 'gold-standard' test for diabetes, is itself < 70% reproducible [32].

**Figure 8:**
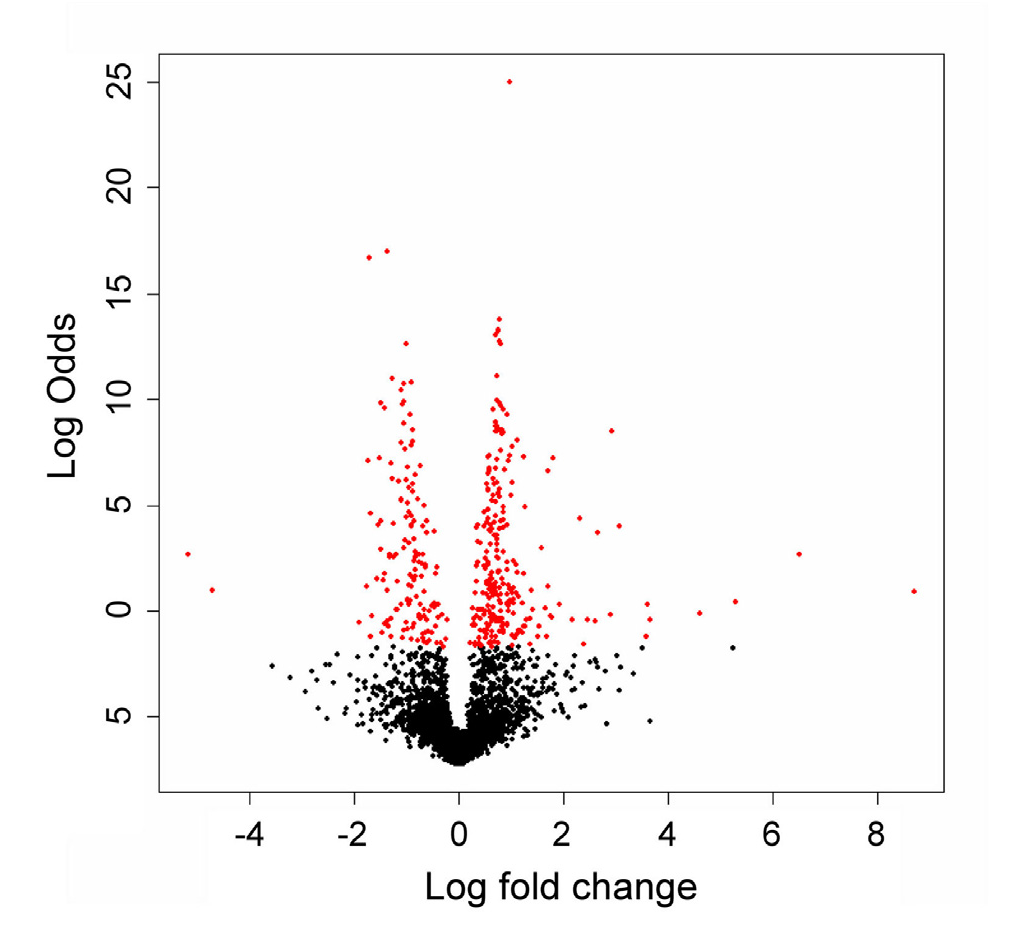
**Corra-generated volcano plot of human type 2 diabetes plasma analyses**. *N*-glycosite peptides were isolated for human plasma samples and analyzed via LC-MS, as described under Methods. Randomized, triplicate analyses were performed for each of 22 human plasma samples, 13 controls (NGT: normal glucose tolerance) and 9 from newly diagnosed cases of type 2 diabetes (DB). Volcano plot displays the 4,240 features that aligned across a minimum of 3 LC-MS runs. The x-axis shows observed log fold change in aligned feature mean intensities between the two sample groups, NGT and DB. The y-axis shows B statistics log Odds for non-random differential abundance obtained for each aligned feature. Red colored dots represent the 400 top-ranked features (in terms of log Odds) that were subsequently targeted for MS/MS identification. A log Odds value of 0 corresponds to a 50% probability non-random differential abundance, and a log Odds of 2.2 to a 90% probability.

An alternate Corra analysis output for this same data is shown in Figure 8. In this case, a "volcano" plot was generated, using LIMMA, to show the detection of differential abundance for the 4240 features that aligned across at least 3 of the 66 LC-MS runs (3 being chosen, in this case, since each individual sample was analyzed in triplicate). In this plot, the aligned features are ordered by log Odds (or B value) on the y-axis. The log Odds B value is, essentially, a measure of probability that the feature is differentially expressed/abundant (as opposed to being observed so by random chance), i.e. the higher the log Odds for each feature, the higher the probability that the feature is genuinely differentially expressed/abundant.

We then ranked these 4240 aligned features according to log Odds for differential abundance to generate a list of the top 400 for targeted MS/MS, i.e. to identify the peptides, hence proteins, that best discriminated between the NGT and DB diagnostic groups. An inclusion list was thus made for these 400 features and two samples, each a pool of 4 randomly chosen plasma isolates for each disease state, NGT and DB, were analyzed, separately, in triplicate, for acquisition of MS/MS data on an FT-LTQ spectrometer. In this way, we were able to assign peptide sequence identity to over half of the 400 targeted features (data not shown). Table 1 shows the top 20 most discriminatory peptides (i.e. highest log Odds, using a cut-off of ≥ 2.5) that were also successfully matched to a peptide sequence, with a PeptideProphet score of ≥ 0.9 via MS/MS, which corresponded to a false discovery rate of 2%. A log Odds of 2.5 represents a minimum probability of 92.4% for likelihood of non-random differential abundance for these 20 peptides, which, in turn, had a minimum probability 91% for correct identification by MS/MS (as determined by PeptideProphet [29]).

**Table 1:** Top 20 most discriminatory peptides for diabetes identified by follow-up targeted MS/MS analysis.

**B (log Odds)**	**Prob**.	**MS1 *m/z***	**z MS1**	**MS2 *m/z***	**z MS1**	**Peptide Sequence**	**IPI Accession Number**
17.02	0.99	529.27	3	529.27	3	R.THLAPYSDELRQR.L	IPI00021841

16.68	0.99	522.77	2	522.77	2	R.LDVDQALN#R.S	IPI00023019

10.99	1.00	697.81	2	697.82	2	K.AADDTWEPFASGK.T	IPI00022432,IPI00646384

10.76	0.93	1181.57	3	1181.58	3	K.SLGNVN#FTVSAEALESQELCGTEVPSVPEHGRK.D	IPI00478003

9.75	1.00	820.90	2	820.91	2	K.AGLQAFFQVQECN#K.S	IPI00017601,IPI00794184

8.63	0.95	730.85	2	730.85	2	K.NLFLN#HSEN#ATAK.D	IPI00431645,IPI00477597,IPI00478493, IPI00607707,IPI00641737

8.48	0.98	637.86	2	637.87	2	R.LLVVYPWTQR.F	IPI00217471,IPI00220706,IPI00473011, IPI00554676,IPI00654755,IPI00657660, IPI00657911,IPI00744503,IPI00749035, IPI00784636,IPI00791558,IPI00796636

8.43	0.99	1340.69	2	1340.69	2	K.MVSHHN#LTTGATLINEQWLLTTAK.N	IPI00431645,IPI00477597,IPI00478493, IPI00607707,IPI00641737

8.00	0.99	579.76	2	579.76	2	R.EEQFN#STFR.V	IPI00168728,IPI00399007,IPI00426051, IPI00470657,IPI00472345,IPI00784807, IPI00784942

6.96	1.00	1082.58	2	1082.58	2	K.VSN#QTLSLFFTVLQDVPVR.D	IPI00478003

5.79	1.00	599.34	3	599.34	3	K.VVLHPN#YSQVDIGLIK.L	IPI00431645,IPI00478493,IPI00641737

5.49	1.00	553.30	2	553.30	2	R.GLN#VTLSSTGR.N	IPI00032258,IPI00418163,IPI00643525, IPI00654875

5.29	0.95	1274.91	3	1274.92	3	R.GNEANYYSN#ATTDEHGLVQFSIN#TTNVMGTSLTVR.V	IPI00478003

5.09	1.00	1178.53	2	1178.55	2	R.GLTFQQN#ASSMCVPDQDTAIR.V	IPI00479708,IPI00549291,IPI00748158, IPI00784931

3.95	0.91	758.05	3	758.05	3	K.TVLTPATNHMGN#VTFTIPANR.E	IPI00164623,IPI00783987

3.58	0.99	952.44	2	952.45	2	R.FSDGLESN#SSTQFEVKK.Y	IPI00032258,IPI00418163,IPI00643525, IPI00654875

3.24	0.94	598.77	2	598.78	2	R.DIENFN#STQK.F	IPI00019943

2.82	1.00	799.43	2	799.43	2	V.LHPN#YSQVDIGLIK.L	IPI00431645,IPI00478493,IPI00641737

2.69	1.00	1183.55	2	1183.56	2	K.STGKPTLYN#VSLVMSDTAGTCY.-	IPI00385264,IPI00549291,IPI00748158, IPI00784931,IPI00788597

2.50	1.00	634.29	2	634.29	2	K.LGN#WSAMPSCK.A	IPI00298828

B (log Odds) indicates Odds of the peptide/proteins are differentially expressed between NGT and DB groups. PeptideProphet [29] probability (Prob.) indicates the probability of correctly assigning MS/MS spectra to the given peptide. To be listed here, a peptide first had to score log Odds of ≥ 2.5 for differential abundance, and then ≥ 0.9 by PeptideProphet for subsequent MS/MS peptide sequence assignment. We also give the *m/z *value and charge state (z) for both the feature initially detected by MS1, as well for the peptide subsequently identified by MS/MS (MS2). For peptide sequence identifications, N# is used to indicate the predicted position of the modified asparagine residues, resulting from their conversion to aspartic acid during the deglycosylation step used during sample preparation. The consensus sequence for *N-*glycosylation is -N-X-S/T- where X is any amino acid except proline, and S/T is serine or threonine. The IPI database entries that were matched to the given sequences are also listed. The log Odds B value is a statistical measure of the likelihood that a given feature is genuinely differentially expressed/abundant, where higher log Odds values represent increased probability of such. Log Odds can be converted to a probability. In this case, where we used a log Odds cut-off of ≥ 2.5, the probability that a feature at this value really is differentially abundant is: exp(2.5)/(1+exp(2.5)) = 0.924, (i.e. 92.4% likely that the observed differential abundance was non-random). Thus a log Odds value of 0 corresponds to a 50–50 chance that the feature is genuinely differentially abundant.

Finally, it should be noted here that these data are from a pilot study, and as such, none of the proteins identified were, or should be considered as candidate markers for diabetes without further studies. Nevertheless, they do illustrate how the Corra framework was implemented to determine, and then identify, targets of interest in any LC-MS-based biomarker discovery workflow. In turn, it also shows how Corra could be of use to biologists or other researchers interested in LC-MS data analysis of any other, similar comparison of related physiological states.

### Application of Corra to phosphopeptide profiling of an Ark1 kinase knockout yeast strain

As a second example of Corra application, we analyzed LC-MS profiles of phosphopeptides isolated from a wild-type yeast strain, for comparison to those obtained from a yeast strain lacking the protein kinase Ark1. The goal was to see whether we could identify potential Ark 1 target proteins, and phosphorylation sites, for this kinase. Unlike for the human diabetes pilot study above, here we fully expected to observe the 'missing features' effect in the Ark 1 deletion strain. Additionally, these analyses were performed on a very high mass accuracy OrbiTrap-LTQ spectrometer, and the SuperHirn tool was instead used for the feature picking and alignments within Corra.

We therefore prepared total phosphopeptide isolates from the two yeast strains, as previously described [27] and summarized under Methods. The two samples were then analyzed on an OrbiTrap-LTQ spectrometer, in triplicate (6 LC-MS runs in total), limiting the LC-retention time range for data analysis to the 20 to 90 minute window, since this was the region where the peptides eluted for these analyses. On average, SuperHirn detected ~23,300 features per LC-MS run, with 54,059 total detected features. Of these, 6,840 aligned across all six LC-MS runs, with 22,562 that aligned across three or more LC-MS runs.

Since there were 2 biological samples, each analyzed in triplicate, we took the 22,562 features that aligned across three or more runs for importing into the CorraStatistics.R module, to search for the differentially abundant features between the control and the kinase knockout yeast strains. In this study, we were especially interested in phosphopeptides not detected in the knockout strain *versus *the control strain, since these would represent potential targets for the missing kinase. Thus to do this, we utilized the 'n/a replace' (for missing values) functionality within Corra to set missing features between all 6 runs to the minimum intensity value detected for the entire dataset. While we recognized that this would generate artificial ratios when we performed the analysis, it nevertheless provided us with the information we needed, since we were, ultimately, only interested in peptide identity here, and not a quantitative measure of differential abundance.

**Figure 9:**
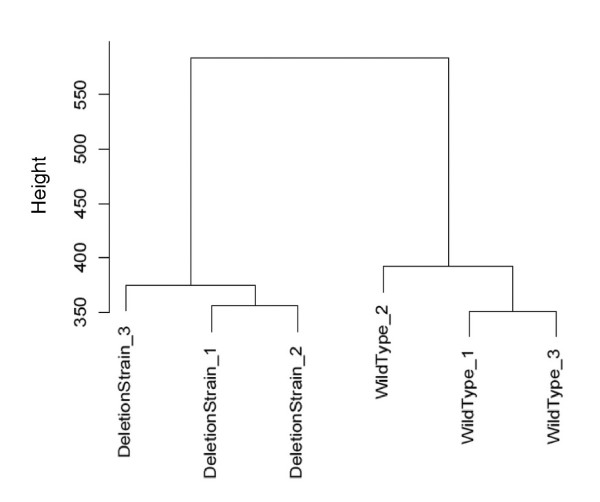
**Corra-generated hierarchical clustering of yeast phosphopeptide analyses**. Phosphopeptides were isolated from two yeast strains, one wild type, and the other an Ark1 protein kinase knockout, and analyzed in triplicate on a very high mass accuracy LC-MS platform, as described under Methods. The 22,562 Corra-detected features that aligned across 3 or more LC-MS runs were used to produce this hierarchical cluster that well distinguished between the two samples, as expected.

**Figure 10:**
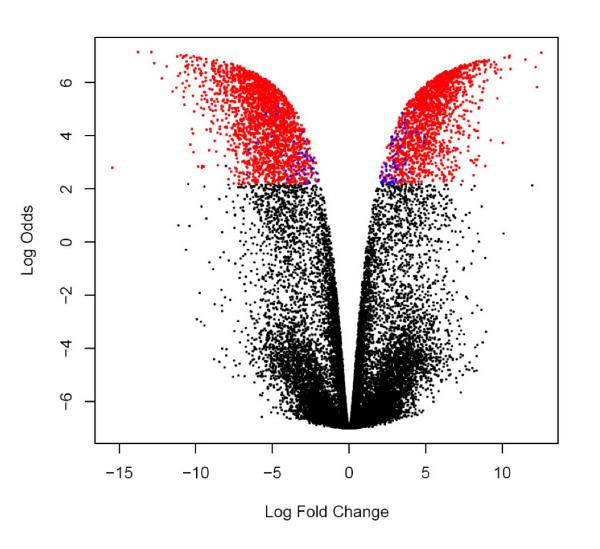
**Corra generated volcano plot of yeast phosphopeptide analyses**. Phosphopeptides were isolated from two yeast strains, one wild type, and the other an Ark1 protein kinase knockout, and analyzed in triplicate on a very high mass accuracy LC-MS platform, as described under Methods. Volcano plot displays 22,562 features that aligned across 3 or more LC-MS runs. The x-axis shows observed log fold change in aligned feature mean intensities between the two yeast strains. The y-axis shows B-statistics log Odds for non-random differential abundance obtained for each aligned feature. Red colored dots indicate features with a log Odds value of ≥ 2.2 (which translates to a posterior probability of 90% chance of non-random differential abundance) and that also utilized the 'n/a replace' capability in Corra (for missing values). Blue colored dots indicate features with a log Odds value of ≥ 2.2, but did not require use of the 'n/a replace' function. A log Odds value of 0 corresponds to a 50% probability of non-random differential abundance, and a log Odds of 2.2 to a 90% probability.

Figure 9 shows a clustering analysis for all 22,562 features that aligned across 3 or more runs, and demonstrated that, as expected, the aligned features distinguished very well between the two yeast strains. The excellent separation observed between the replicate analyses of each sample was clearly enhanced by the large, artificial, ratios generated via use of the 'n/a replace' function. A volcano plot, shown in Figure 10, shows the log Odds distribution for differential abundance, for the same 22,562 features aligned in 3 or more runs. Those with a log Odds value of ≥ 2.2 (i.e. > 90.0% chance of non-random observation of differential abundance), and for which the 'n/a replace' function was used, are colored red. The smaller number of blue-colored features represent those also with a log Odds value of ≥ 2.2, but for which the 'n/a replace' was not required, and thus these generally showed lower ratios of differential abundance (i.e. not artificial) than the red-colored features. In comparing Figures 8 and 10, we can also make a couple of general observations. In the yeast study, we observed much larger ratios, almost certainly due to the replacement of missing features. On the other hand, in the human diabetes study shown in Figure 8, we observed much larger log Odds values (i.e. increased confidence in differential abundance). This is almost certainly due to the much larger sample size (66 LC-MS analyses *vs*. 6 in the yeast study), therefore leading to better statistical confidence.

**Figure 11:**
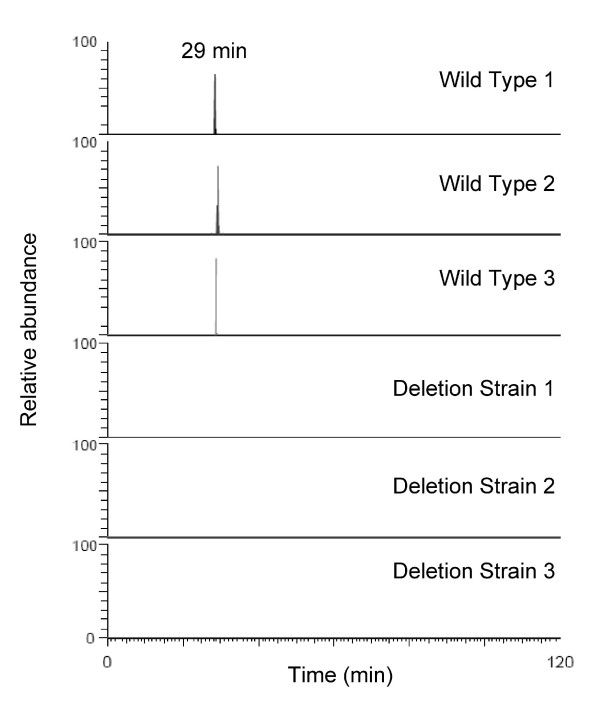
**Verification of a Corra-identified Ark1 kinase substrate peptide/protein**. Following targeted MS/MS identification of the top-ranked Corra-identified discriminatory features (see Figure 10 and Table 2) ion chromatograms were extracted from all LC-MS runs for the peptide RHS*LGLNEAKK (*m/z *= 444.895 [M+3H]^3+^), where S* represents phosphoserine. This peptide was derived from the protein YDR293C, and was confirmed as present in all 3 control sample analyses, but absent in all 3 Ark1 knockout analyses, as would be expected. For all six plots, a relative abundance of 100% was manually set to 10^7 ^ion counts so that all were on the same scale.

From these data analyses, as with the diabetes study above, we next made an inclusion list for targeted MS/MS, to try and identify some of the phosphopeptides lost in the Ark1 knockout yeast *versus *the control. Table 2 lists the top 12 most discriminatory peptides, with a log Odds of ≥ 2.2, and that also matched a peptide sequence by MS/MS, with a PeptideProphet score of ≥ 0.7 (representing a false discovery rate of 5%). Ark1 is known to be involved in endocytosis and actin reorganization, as also are 4 other proteins from Table 2 (YOL109W, YBL037W, YMR109W, and YJR083C), demonstrating that Corra successfully enabled the generation of potentially biologically relevant information. Finally, for confirmation purposes, Figure 11 shows extracted ion chromatograms, for all 6 LC-MS runs, for the identified YDR293 peptide, RHS*LGLNEAKK (where S* represents phosphoserine) at *m/z *= 444.895 [M+3H]^3+^, confirming it's detection in all 3 replicate analyses of the control strain, and its absence in all 3 replicate analyses of the Ark1 knockout strain.

**Table 2:** Top 12 most discriminatory yeast phosphopeptides identified by follow-up targeted MS/MS.

**B (log Odds)**	**Prob**.	**MS1 *m/z***	**z MS1**	**MS2 *m/z***	**z MS2**	**Peptide Sequence**	**SGD ID**	**Protein**
6.45	1.00	874.74	3	875.25	3	EQAEASIDNLKNEAT*PEAEQVKK	S000005469	YOL109W

6.45	0.91	874.74	3	875.22	3	RYSNTT*INNANGGTSAGSTTGAALSR	S000001397	YIL135C

6.20	0.88	546.27	3	546.61	3	KMS*FSGYSPKPISK	S000005701	YOR175C

6.19	0.73	994.82	3	995.40	3	LGSLPASAGSTTLINTPSEASSS*TPDLLSK	S000000133	YBL037W

6.09	1.00	459.91	3	460.18	3	RPS*VIGFLSGHK	S000000197	YBL101C

6.04	0.93	1152.90	3	1153.57	3	SNGAPTNAS*PAPPPVILQPTLQPNDQGNPLNTR	S000000316	YBR112C

5.97	0.99	411.54	3	445.15	3	RHS*LGLNEAKK	S000002701	YDR293C

5.94	0.99	637.00	3	637.37	3	RPVS*IAAAQHVPTAPASR	S000004715	YMR109W

5.48	0.99	938.44	3	938.98	3	S*INDFFSKPNNNVNSNINNTLPNR	S000003843	YJR083C

5.45	0.94	840.91	2	841.37	2	TTS*LVNNILNGNNAR	S000004748	YMR140W

5.26	0.91	1068.77	3	1069.39	3	QQHRPVPATSAGEPTDETEPES*DTEVETS	S000005609	YOR083W

4.33	0.94	754.66	3	663.72	3	RSS*GPMDFQNTIHNMQYR	S000001913	YFR017C

B (log Odds) indicates Odds of the peptide/proteins are differentially expressed between wild type yeast and deletion strain group. PeptideProphet [29] probability (Prob.) indicates the probability of correctly assigning MS/MS spectra to the given peptide. We also give the *m/z *value and charge state (z) for both the feature initially detected by MS1, as well for the peptide subsequently identified by MS/MS (MS2). For peptide sequence identifications, S* and T* indicate the predicted position of the modified (phosphorylated) serine or threonine residues reported by SEQUEST search and manual/visual inspection of the MS/MS spectra. The yeast SDG database accession numbers matched to the sequences are also listed, along with their corresponding SGD systematic protein names. The log Odds B value is a statistical measure of the likelihood that a given feature is genuinely differentially expressed/abundant, where higher log Odds values represent increased probability of such. Log Odds can be converted to a probability. In this case, a log Odds of 4 corresponds exp(4)/(1+exp(4)) = 0.982 (i.e. 98.2% likely that the observed differential abundance was non-random).

## Conclusion

Here we present Corra, a complete, free and open source computational platform that enables LC-MS-based proteomic workflows, and as such, addresses an unmet need in the LC-MS proteomics field. The Corra framework extends and adapts existing algorithms used for LC-MS-based proteomics, as well as existing statistical algorithms from the microarray community suitable for the analysis of high-dimensional LC-MS data, with a view to controlling false-discovery rates. Corra also adapts additional software engineering technologies, such as distributed processing and Google Web Toolkit. Corra enables the use of an array of LC-MS data alignment tools, integrating them with various forms of statistical analyses via Bioconductor, for the generation of statistically validated lists of differentially abundant peptide features for subsequent targeted LC-MS/MS identification, all within a single computational platform. Corra is not intended as an alternative to pre-existing LC-MS analytical tools, such as SpecArray [12], SuperHirn [15], msInspect [8,13], PEPPeR [10], and others, but rather provides a framework for using such tools in a way that can overcome some of their limitations, such as the ability to process datasets of large size, via Corra's distributed computation process. Corra effectively obviates cross-tool incompatibilities through the use of a new common data format, AMPL, that allows for rich data annotation from multiple experimental workflows, as well as a set of parsers between different data input/output formats, including R's ExpressionSet format for statistical data analysis. Finally, in part through its implementation via a web-based GUI, Corra fills a noticeable gap in the field of LC-MS, or 'label-free' quantitative proteomics, in that it provides for biologists and other researchers, not just those trained in bioinformatics, to process, visualize and analyze their data in the manner of their choosing, all within a single application, and on their own workstation.

In the version of Corra presented here, two of the aforementioned LC-MS tools, SpecArray and SuperHirn were implemented, as were the Bioconductor packages LIMMA and maSigPro for statistical analysis. However, with the description of the a common file format for LC-MS data, APML, and with the parsers and documentation also provided with Corra, the ability to incorporate additional software tools and statistical algorithms for additional workflows is fully supported. This allows for a user with appropriate bioinformatics training to set up Corra on any Linux server, either as is, or customized for their own personal or groups workflows. Finally, Corra is provided as open source (Apache 2.0), and may be downloaded, along with all relevant documentation [17]. Corra is also available from Sourceforge.net (keyword search: Corra). Further development of Corra and APML remain ongoing. Upcoming releases of Corra will include an APML-ready version of the LC-MS analysis tool msInspect [8], additional functionality to allow for the extraction and quantification of LC-MS data that includes the incorporation of stable isotope labels, and providing Corra outputs in a format suitable for downstream interaction with selected reaction monitoring-based workflows.

## Abbreviations

APML: annotated putative peptide markup language; DB: type 2 diabetes; ESI: electrospray ionization; FT: Fourier transform; Glufib: glu1-fibrinopeptide B; GUI: graphical user interface; LC-MS: liquid chromatography mass spectrometry; LIMMA: linear models for microarray data; MS/MS: tandem mass spectrometry; NGT: normal glucose tolerance; OGTT: oral glucose tolerance test; PNGaseF: protein *N*-glycosidase F; SD: synthetic defined; TOF: time of flight; XML: extensible markup language.

## Authors' contributions

MB, DC, JE, AG, OV and NZ conceived the original Corra framework, and MB subsequently oversaw Corra framework design, including APML and data analysis tools, and their implementation. MB amended and revised the SpecArray tool for implementation within Corra. LM wrote the SuperHirn software tool and collaborated with MB for modifications required for Corra implementation. VS assisted with Corra data analyses and designed and implemented the Corra GUI. OV did the initial design for the statistical analytical methods. BB performed the yeast phosphopeptide experiments and preliminary data analyses. KC performed glycopeptide isolations for the human diabetes plasma study. HL performed the LC-MS analyses for the human diabetes plasma study. SL oversaw all mass spectrometry experiments at ISB and performed targeted MS/MS experiments for the human diabetes plasma study. RA oversaw the research contributed by ETH and designed the yeast phosphopeptide study with BB. JW oversaw the research contributed by ISB and designed the human diabetes plasma study. JW and MB wrote the manuscript.

## References

[B1] Aebersold R, Mann M (2003). Mass spectrometry-based proteomics. Nature.

[B2] Gillette MA, Mani DR, Carr SA (2005). Place of pattern in proteomic biomarker discovery. J Proteome Res.

[B3] MacCoss MJ, Matthews DE (2005). Quantitative MS for proteomics: teaching a new dog old tricks. Anal Chem.

[B4] Mueller LN, Brusniak MY, Mani DR, Aebersold R (2008). An assessment of software solutions for the analysis of mass spectrometry based quantitative proteomics data. J Proteome Res.

[B5] Gygi SP, Rist B, Gerber SA, Turecek F, Gelb MH, Aebersold R (1999). Quantitative analysis of complex protein mixtures using isotope-coded affinity tags. Nat Biotechnol.

[B6] Ross PL, Huang YN, Marchese JN, Williamson B, Parker K, Hattan S, Khainovski N, Pillai S, Dey S, Daniels S (2004). Multiplexed protein quantitation in Saccharomyces cerevisiae using amine-reactive isobaric tagging reagents. Mol Cell Proteomics.

[B7] Ong SE, Blagoev B, Kratchmarova I, Kristensen DB, Steen H, Pandey A, Mann M (2002). Stable isotope labeling by amino acids in cell culture, SILAC, as a simple and accurate approach to expression proteomics. Mol Cell Proteomics.

[B8] Bellew M, Coram M, Fitzgibbon M, Igra M, Randolph T, Wang P, May D, Eng J, Fang R, Lin C (2006). A suite of algorithms for the comprehensive analysis of complex protein mixtures using high-resolution LC-MS. Bioinformatics.

[B9] Du P, Sudha R, Prystowsky MB, Angeletti RH (2007). Data reduction of isotope-resolved LC-MS spectra. Bioinformatics.

[B10] Jaffe JD, Mani DR, Leptos KC, Church GM, Gillette MA, Carr SA (2006). PEPPeR, a platform for experimental proteomic pattern recognition. Mol Cell Proteomics.

[B11] Katajamaa M, Miettinen J, Oresic M (2006). MZmine: toolbox for processing and visualization of mass spectrometry based molecular profile data. Bioinformatics.

[B12] Li XJ, Yi EC, Kemp CJ, Zhang H, Aebersold R (2005). A software suite for the generation and comparison of peptide arrays from sets of data collected by liquid chromatography-mass spectrometry. Mol Cell Proteomics.

[B13] May D, Fitzgibbon M, Liu Y, Holzman T, Eng J, Kemp CJ, Whiteaker J, Paulovich A, McIntosh M (2007). A platform for accurate mass and time analyses of mass spectrometry data. J Proteome Res.

[B14] Mayr BM, Kohlbacher O, Reinert K, Sturm M, Gropl C, Lange E, Klein C, Huber CG (2006). Absolute myoglobin quantitation in serum by combining two-dimensional liquid chromatography-electrospray ionization mass spectrometry and novel data analysis algorithms. J Proteome Res.

[B15] Mueller LN, Rinner O, Schmidt A, Letarte S, Bodenmiller B, Brusniak MY, Vitek O, Aebersold R, Muller M (2007). SuperHirn – a novel tool for high resolution LC-MS-based peptide/protein profiling. Proteomics.

[B16] Smith CA, Want EJ, O'Maille G, Abagyan R, Siuzdak G (2006). XCMS: processing mass spectrometry data for metabolite profiling using nonlinear peak alignment, matching, and identification. Anal Chem.

[B17] Seattle Proteome Center (SPC) – Corra. http://tools.proteomecenter.org/Corra/corra.html.

[B18] Desiere F, Deutsch EW, Nesvizhskii AI, Mallick P, King NL, Eng JK, Aderem A, Boyle R, Brunner E, Donohoe S (2005). Integration with the human genome of peptide sequences obtained by high-throughput mass spectrometry. Genome Biol.

[B19] Zhang H, Loriaux P, Eng J, Campbell D, Keller A, Moss P, Bonneau R, Zhang N, Zhou Y, Wollscheid B (2006). UniPep–a database for human N-linked glycosites: a resource for biomarker discovery. Genome Biol.

[B20] Bioconductor: open source software for bioinformatics. http://www.bioconductor.org/.

[B21] Smyth GK (2004). Linear models and empirical bayes methods for assessing differential expression in microarray experiments. Stat Appl Genet Mol Biol.

[B22] Conesa A, Nueda MJ, Ferrer A, Talon M (2006). maSigPro: a method to identify significantly differential expression profiles in time-course microarray experiments. Bioinformatics.

[B23] Keller A, Eng J, Zhang N, Li XJ, Aebersold R (2005). A uniform proteomics MS/MS analysis platform utilizing open XML file formats. Mol Syst Biol.

[B24] Proteomic and metabolomic approaches to diagnose diabetes and pre-diabetes. http://grants.nih.gov/grants/guide/pa-files/PAR-04-076.html.

[B25] Zhou Y, Aebersold R, Zhang H (2007). Isolation of N-linked glycopeptides from plasma. Anal Chem.

[B26] Schmidt A, Gehlenborg N, Bodenmiller B, Mueller LN, Campbell D, Mueller M, Aebersold R, Domon B (2008). An integrated, directed mass spectrometric approach for in-depth characterization of complex peptide mixtures. Mol Cell Proteomics.

[B27] Bodenmiller B, Mueller LN, Mueller M, Domon B, Aebersold R (2007). Reproducible isolation of distinct, overlapping segments of the phosphoproteome. Nat Methods.

[B28] Bodenmiller B, Mueller LN, Pedrioli PG, Pflieger D, Junger MA, Eng JK, Aebersold R, Tao WA (2007). An integrated chemical, mass spectrometric and computational strategy for (quantitative) phosphoproteomics: application to Drosophila melanogaster Kc167 cells. Mol Biosyst.

[B29] Keller A, Nesvizhskii AI, Kolker E, Aebersold R (2002). Empirical statistical model to estimate the accuracy of peptide identifications made by MS/MS and database search. Anal Chem.

[B30] Pedrioli PG, Eng JK, Hubley R, Vogelzang M, Deutsch EW, Raught B, Pratt B, Nilsson E, Angeletti RH, Apweiler R (2004). A common open representation of mass spectrometry data and its application to proteomics research. Nat Biotechnol.

[B31] Gentleman R, Carey V, Huber W, Irizarry R, Dudoit S (2005). Bioinformatics and Computational Biology Solutions Using R and Bioconductor.

[B32] Ko GT, Chan JC, Woo J, Lau E, Yeung VT, Chow CC, Cockram CS (1998). The reproducibility and usefulness of the oral glucose tolerance test in screening for diabetes and other cardiovascular risk factors. Ann Clin Biochem.

